# Supplementary figures and images for: Silencing of lncRNA AK045490 Promotes Osteoblast Differentiation and Bone Formation via β-Catenin/TCF1/Runx2 Signaling Axis
Source: Int J Mol Sci. 2019 Dec 10;20(24):6229. doi: 10.3390/ijms20246229 (PMC6941011; doi:10.3390/ijms20246229)

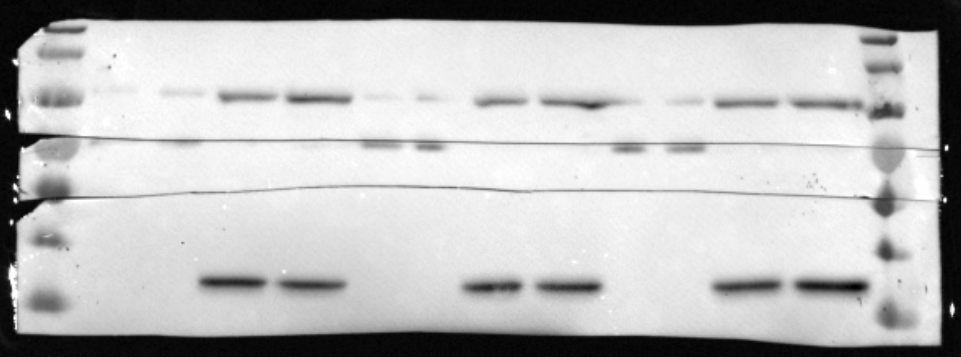

Supplement: Supplementary file 1 [file ijms-20-06229-s001.zip › Figure 3b Supplement-WB-(A)-v1.tif]

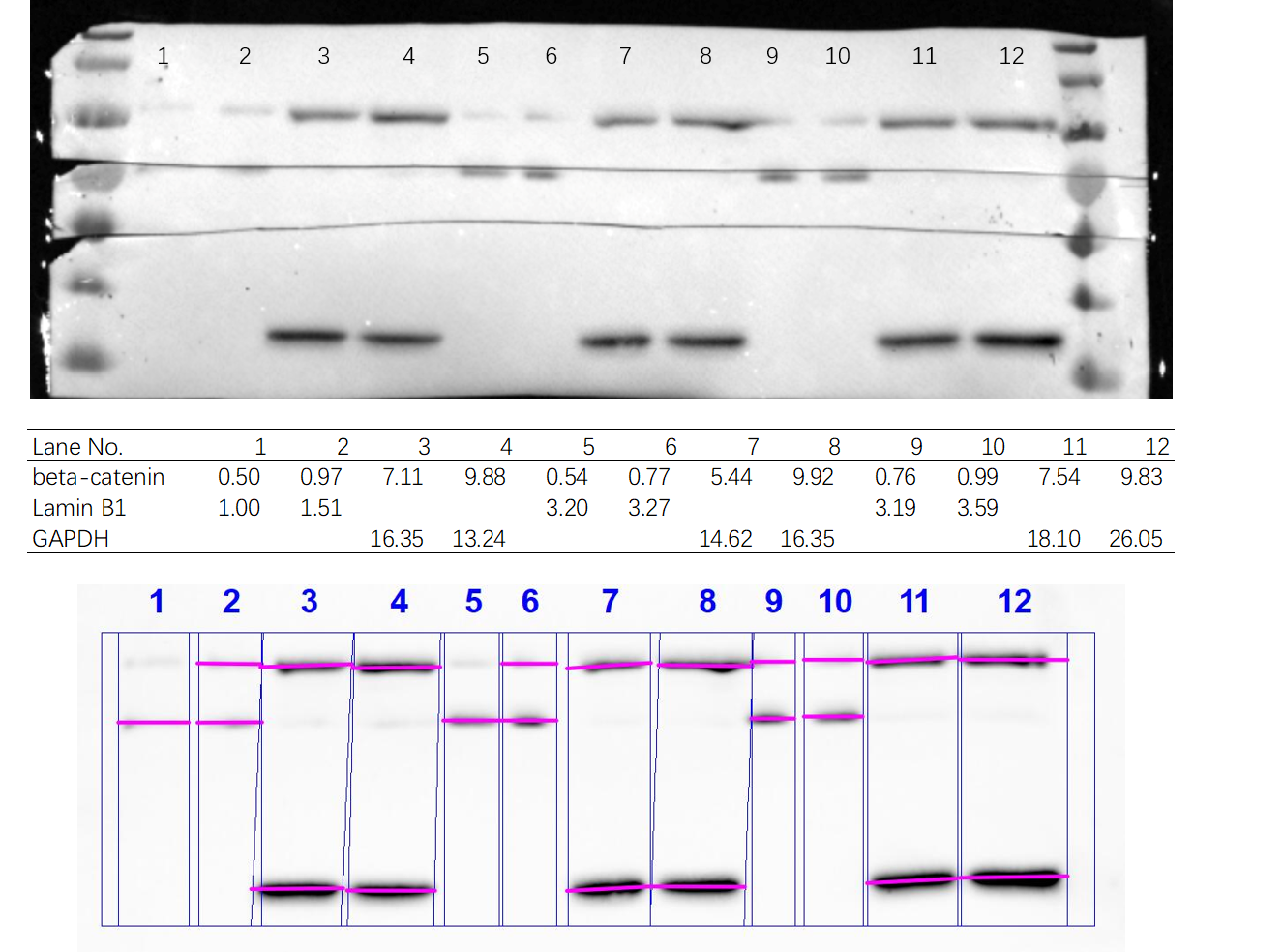

Supplement: Supplementary file 1 [file ijms-20-06229-s001.zip › Figure 3b Supplement-WB-(A)-v3.tif]
